# An Electrochemical Molecularly Imprinted Polymer Sensor for Rapid β-Lactoglobulin Detection

**DOI:** 10.3390/s21248240

**Published:** 2021-12-09

**Authors:** Bixuan Wang, Jingyi Hong, Chun Liu, Liying Zhu, Ling Jiang

**Affiliations:** 1 School of Chemistry and Molecular Engineering, Nanjing Tech University, Nanjing 211816, China; 201961105021@njtech.edu.cn; 2 State Key Laboratory of Materials-Oriented Chemical Engineering, College of Food Science and Light Industry, Nanjing Tech University, Nanjing 211816, China; hjy1364828909@outlook.com (J.H.); 201961219027@njtech.edu.cn (C.L.); jiangling@njtech.edu.cn (L.J.)

**Keywords:** β-lactoglobulin, molecularly imprinted polymer, electrochemical sensor, screen-printed carbon electrode

## Abstract

Facile detection of β-lactoglobulin is extraordinarily important for the management of the allergenic safety of cow’s milk and its dairy products. A sensitive electrochemical sensor based on a molecularly imprinted polymer-modified carbon electrode for the detection of β-lactoglobulin was successfully synthesized. This molecularly imprinted polymer was prepared using a hydrothermal method with choline chloride as a functional monomer, β-lactoglobulin as template molecule and ethylene glycol dimethacrylate as crosslinking agent. Then, the molecularly imprinted polymer was immobilized on polyethyleneimine (PEI)-reduced graphene oxide (rGO)-gold nanoclusters (Au-NCs) to improve the sensor’s selectivity for β-lactoglobulin. Under optimal experimental conditions, the designed sensor showed a good response to β-lactoglobulin, with a linear detection range between 10^−9^ and 10^−4^ mg/mL, and a detection limit of 10^−9^ mg/mL (S/N = 3). The developed electrochemical sensor showed a high correlation in the detection of β-lactoglobulin in four different milk samples from the market, indicating that the sensor can be used with actual sample.

## 1. Introduction

Milk and dairy products are an important part of the human diet, owing to their rich nutrition and easy absorption. However, some people are allergic to milk and dairy products because of the presence of allergenic proteins, which can make some people face serious adverse reactions in the skin, gastrointestinal tract, or respiratory tract [1,2,3]. Avoidance of allergenic food is an efficient management method, but milk is the only food for infants. Accordingly, to protect the allergic population and still satisfy their need for milk, a special formula without allergenic proteins has been developed [4]. As a result, the detection for allergenic proteins has become essential to ensure the safety of hypoallergenic milk.

Beta-lactoglobulin (β-Lg) is a major allergen in milk, since it accounts for 10% of the total milk protein and 60% of the total whey protein in milk (2–3 g/L in ordinary milk) [5]. In order to protect allergic people, the protein threshold value was set to 0.1 mg/mL [6]. Due to the presence of other proteins, selective and sensitive analytical methods are essential for the quantification of β-Lg. The reported methods for β-Lg detection include the enzyme-linked immunosorbent assay (ELISA) [7], high performance liquid chromatography (HPLC) [8], capillary electrophoresis (CE) [9], and surface plasmon resonance (SPR) [10]. Nevertheless, all these methods have multiple disadvantages, such as complicated sample processing, long analytical time, and expensive reagents.

In recent years, electrochemical sensors are increasingly being used for food allergen detection, due to their high sensitivity, simple operation, and low cost [11]. However, the accuracy of results determined by electrochemical sensors tends to be influenced by the complicated components in milk. One approach to overcome this problem is to develop molecularly imprinted polymers (MIPs) to selectively distinguish β-Lg from other components [12]. MIP are synthesized according to a particular template molecule, which can be recognized selectively among other components of the sample matrix [13]. Molecular imprinting technology has a lot of advantages, such as high binding capacity, high mass transfer rate towards targets [14,15,16], specific recognition, and wide practicability. Currently, MIP screen-printed electrode sensors are widely used in electrochemical detection. For example, Ding et al. used molecularly imprinted polypyrrole nanotubes to detect glyphosate [17]. Ekomo et al. used electroactive molecularly imprinted polymer to detect bisphenol A [18]. Additionally, Motia et al. developed a sensor for detecting glycerin based on MIP [19]. However, some drawbacks of MIP, such as incomplete template removal and weak conductivity, may limit their application in the sensor field [16,20]. Thus, the related effects of conductive materials should be considered for obtaining high-sensitivity sensors. To improve electrical conductivity, researchers have reported a number of materials for electrochemical detection to enhance imprinting sites and electron transfer efficiency, including three-dimensional carbon nanotubes [21], functionalized multi-walled carbon nanotubes [22], ferrocene [23], and ferroferric oxide [16]. Recently, graphene is increasingly considered an ideal carrier for unlabeled electrochemical sensors because of its excellent electrical and mechanical properties [24]. To avoid the loss of electrochemically active regions, graphene (GO) and reduced graphene (rGO) are often combined with different nanomaterials, such as gold nanoparticles, polyaniline, carbon nanotubes, chitosan, and methylene green to increase the sensitivity of the sensor [25]. In particular, rGO-supported gold nanoclusters offer greatly increased conductivity due to increased charge transfer from the base to the nanoclusters. Stable nanocomposites can also be assembled through the electrostatic interaction of gold nanoclusters and PEI-rGO [26].

In order to develop a simple sensor with high selectivity and sensitivity for the detection of β-Lg, PEI-rGO-Au-NCs@MIP composites were prepared. We used β-Lg as template for hydrothermal synthesis of MIP as an alternative to expensive β-Lg antibodies, and immobilized MIP on PEI-rGO-Au-NCs composites. The current change caused by the reaction of MIP with β-Lg, as well as the electrochemical performance of the sensor, was monitored to evaluate the results. After modification, the electrochemical sensors showed an obvious improvement in the selectivity and electrical signal in response to β-Lg. Furthermore, under the optimized conditions, the PEI-rGO-Au-NCs@MIP exhibited satisfied selectivity, good linearity, and a low detection limit for β-Lg. As a new type of electrochemical sensor with excellent sensitivity for β-Lg, it can potentially be applied to determine the content of β-Lg in real-world samples.

## 2. Materials and Methods

### 2.1. Chemicals and Reagents

Choline chloride (ChCl), acrylic acid (AA), ethylene glycol dimethacrylate (EDMA), benzoyl peroxid, N-hydroxysuccinimide (NHS), poly acetylimide (PEI), hydrazine hydrate, glutathione (GSH), N,N-dimethylaniline (DMA), and 1-(3-dimethylaminopropyl)-3-ethylcarbodiimide hydrochloride (EDC) were supplied by Aladdin Reagent Co., Ltd. (Shanghai, China). The β-Lg was obtained from Sigma Aldrich Co., Ltd. (St. Louis, MO, USA). Graphene oxide was purchased from Suzhou Tanfeng Graphene Technology Co., Ltd. (Suzhou, China). Chloroauric acid (HAuCl_4_) was offered by Sinopharm Chemical Reagent Co., Ltd. (Shanghai, China) Screen-printed carbon electrode (SPCE) were commercially supplied by Yi Yue Co., Ltd., (Qingdao, China). All aqueous solutions were prepared with deionized water, and all other chemical used in this study were analytical grade.

### 2.2. Instruments

The surface morphology of the synthesized materials was observed using Quanta FEG 250 scanning electron microscope (SEM; FEI, Tokyo, Japan) and a Jem-2100F transmission electron microscope (TEM; Jeol, Tokyo, Japan). The elemental mapping of the synthesized materials was conducted using Quanta FEG 250 EDS mapping (FEI, Japan). The XRD spectra were observed on a powder X-ray diffractometer (Rigaku RINT 2500, Rigaku Corporation, Tokyo, Japan). Infrared spectra were recorded using a Nicolet iS50 Fourier transform infrared spectrometer (Thermo Fisher Scientific Inc., Waltham, MA, USA). Electrochemical measurements were conducted on a CHI 760E electrochemical workstation (Shanghai Chenhua Instruments Co., Shanghai, China). 

### 2.3. Sensor Fabrication

#### 2.3.1. Preparation of PEI-rGO-Au-NCs

The PEI-rGO-Au-NCs were prepared according to our previous report [27]. Briefly, GSH-modified Au-NCs and PEI-rGO were synthesized separately. Then, the synthetic PEI-rGO was dispersed in 40 mL of deionized water. Excess GSH-modified Au-NCs were added to the PEI-rGO solution. After 3 min of sonication and 5 min of 3000× *g* centrifugation, the PEI-rGO-Au-NCs was obtained by discarding the supernatants [28,29,30].

#### 2.3.2. Preparation of Imprinted Polymers

Choline chloride (ChCl, 13.9 mg) and acrylic acid (AA, 14.4 mg) were heated in a water bath at 90 °C until a transparent liquid formed. Then, the liquid was cooled to room temperature to obtain a ChCl-AA deep eutectic solvent (DES), which was maximized with ethylene glycol dimethyl acrylic (EDMA, 59.4 mg) in a test tube. The mixture was sonicated for 5 min after the addition of PEI-rGO-Au-NCs (2 mg). Subsequently, β-Lg and N,N-dimethyl aniline (DMA, 30 μL) were added, followed by soaking in sulfuric acid (10%). After that, the PEI-rGO-Au-NCs@MIP was eluted with ethanol three times to remove the template proteins. Non-imprinted polymers (NIP) and PEI-rGO-Au-NCs@NIP were produced under identical conditions in the absence of β-Lg.

#### 2.3.3. Construction of the Electrochemical Sensor

The surface of the working electrode was covered with 30 μL of PEI-rGO-Au-NCs@MIP and then placed in an oven at 60 °C for 20 min. The obtained electrode was then rinsed three times with phosphate-buffered solution (PBS, 0.1 mol/L, pH 7.0) and stored at 4 °C for further use.

#### 2.3.4. Detection of β-lactoglobulin

For the purpose of β-Lg measurement, 3 μL samples comprising β-Lg of different concentrations were pipetted onto the electrode and incubated at 25 °C for 45 min. The electrochemical sensors were then washed with PBS (0.1 mol/L, pH 7.0) three times before the measurements. Electrochemical measurements such as cyclic voltammetry (CV, potential range: −0.1 to 0.7 V, scan rate: 50 mV/s), differential pulse voltammetry (DPV, potential range: −0.1 to 0.7 V, amplitude: 50 mV, pulse width: 0.05 s, pulse period: 0.5 s, pulse cycle: 0.2 s) and electrochemical impedance spectroscopy (EIS) were carried out in PBS (5.0 mmol/L K_3_ [Fe(CN)_6_], 1.0 mmol/L K_4_ [Fe(CN)_6_], 0.1 mol/L KCl, pH 7.0).

#### 2.3.5. Analysis of Real Samples

Four different brands of milk, including Jindian, Mengniu, Yili, and Telunsu were purchased from the local supermarket in China and used as real-world samples in this work. The milk samples were mixed with deionized water at a dilution ratio of 1:10,000 and centrifuged at 20,000× *g* for 15 min. The supernatants were collected and stored at −20 °C. For comparison, the samples and five standard solutions (12.5, 25, 50, 100, 200 μg/mL) were tested using the β-Lg ELISA system kit. For detection with electrochemical sensors, the milk samples were diluted before analysis as follows: a 100-μL extraction sample was diluted 10 times and incubated for 45 min at 25 °C with the sensors to record the DPV current responses.

## 3. Results and Discussion

### 3.1. Strategy of the Electrochemical Immunoassay

**Figure 1:**
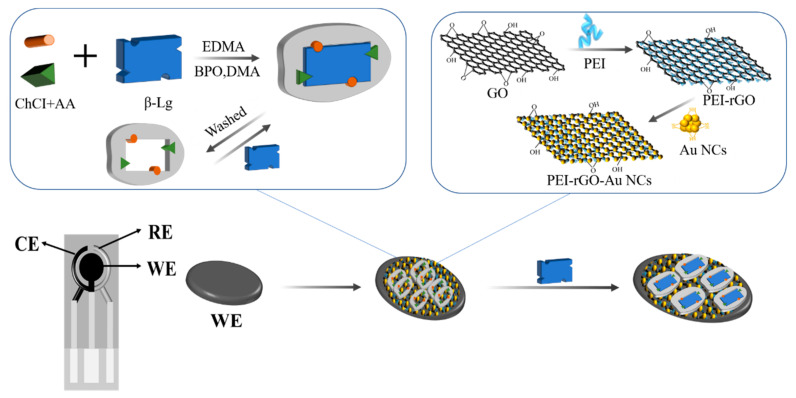
Assembly process of the composite material.

A sensitive electrochemical sensor was developed using PEI-rGO-Au-NCs@MIP as electrode modification material. As shown in Figure 1, we used DES as the functional monomer, β-Lg as the template molecule, EDMA as the crosslinking agent, benzoyl-N-N-dimethylaniline (BPO-DMA) as the initiator, and PEI-rGO-Au-NCs as the composite material. A large number of active sites on the surface of Au-NCs, which were beneficial for the formation of imprinted holes. At the same time, the electrochemical performance of the electrodes can be enhanced by PEI-rGO-Au-NCs. After the elution of the PEI-rGO-Au-NCs@MIP composite material with an organic solvent, the template molecules will be eliminated and a large number of imprints will be produced. These imprints will reabsorb the template molecules when they encounter them again. In this study, disposable screen-printed electrodes were used, including counter electrode (CE), reference electrode (RE), and working electrode (WE). The current signal could be amplified by immobilizing PEI-rGO-Au-NCs@MIP on the working electrode [31,32]. The change of current following the readsorption of template molecules on MIP was recorded to detect β-Lg.

### 3.2. Characterization of Modified Electrodes

**Figure 2:**
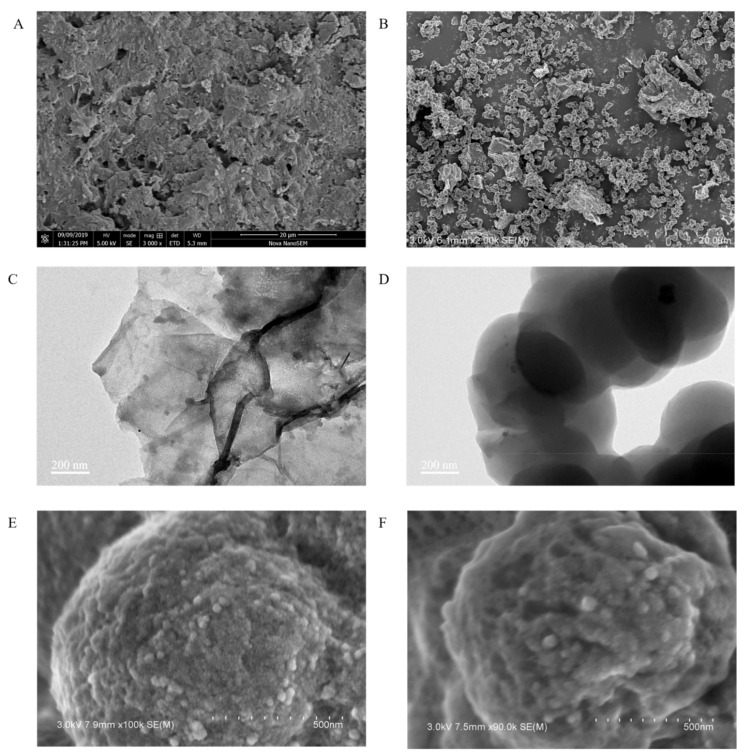
The morphology of the composite materials (**A**,**B**,**E**,**F**): SEM images of PEI-rGO-Au-NCs, PEI-rGO-Au-NCs@MIP, PEI-rGO-Au-NCs@NIP, and PEI-rGO-Au-NCs@MIP; (**C**,**D**): TEM images of PEI-rGO-Au-NCs and MIP).

SEM and TEM were used to observe the morphology and microstructure of PEI-rGO-Au-NCs, PEI-rGO-Au-NCs@MIP and PEI-rGO-Au-NCs@NIP. The overall morphology of PEI-rGO-Au-NCs can be seen in Figure 2A. The surface of the PEI-rGO-Au-NCs material presented a layered structure, which was consistent with a stack of PEI-grafted rGO with Au-NCs on the surface. Compared with PEI-rGO-Au-NCs@NIP (Figure 2E), the PEI-rGO-Au-NCs@MIP was shown in Figure 2F had a rougher surface after elution of the template protein, which be caused by the formation of imprinting cavities. According to the SEM and TEM images, it is obvious that the MIPs were successfully polymerized on the surface of PEI-rGO-Au-NCs.

**Figure 3:**
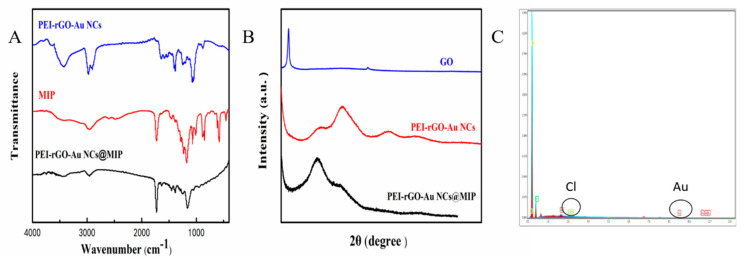
Characterization of PEI-rGO-Au-NCs@MIP (**A**): FTIR spectra; (**B**): XRD pattern; (**C**): EDS.

The structure of PEI-rGO-Au-NCs@MIP was characterized by FTIR, XRD, and EDS. The FTIR results show that the characteristic peaks of PEI-rGO-Au-NCs and MIP could also be found in PEI-rGO-Au-NCs@MIP. As shown in Figure 3A, there was a –OH peak at 3408 cm^−1^, which is similar to that of PEI-rGO-Au-NCs [33]. In addition, the characteristic peaks of C-N (1170 cm^−1^) and C=O (1722 cm^−1^) in MIP are clearly visible. According to the FTIR spectra, both PEI-rGO-Au-NCs and MIP were present in the synthesized material. Figure 3B depicts the XRD patterns of GO, PEI-rGO-Au-NCs and PEI-rGO-Au-NCs@MIP. The GO sample shows a very sharp high strength peak at 10.3°, corresponding to the (002) surface of the graphene sheet. However, the XRD pattern of the PEI-rGO-Au-NCs@MIP sample exhibited a shift in the peak towards 2θ = 22.3°, indicating that GO has been reduced to rGO [34]. The characteristic peaks at 31.0°, 49.0°, and 63.0° correspond to the diffraction crystal plane (111), (200) and (220) of Au (JCPDS04-0784) [35] in the material. The curves of PEI-rGO-Au-NCs@MIP still contain characteristic peaks of PEI-rGO-Au-NCs, implying that the structure of PEI-rGO-Au-NCs was not damaged during the synthesis of MIPs. As shown in Figure 3C, carbon is the major element in this material, with small amounts of Au and Cl. Carbon is mainly derived from the reduced GO, Au from gold nanoclusters, and Cl from choline chloride. All these results suggested that the PEI-rGO-Au-NCs@MIP has been successfully prepared.

### 3.3. Electrochemical Characterization

**Figure 4:**
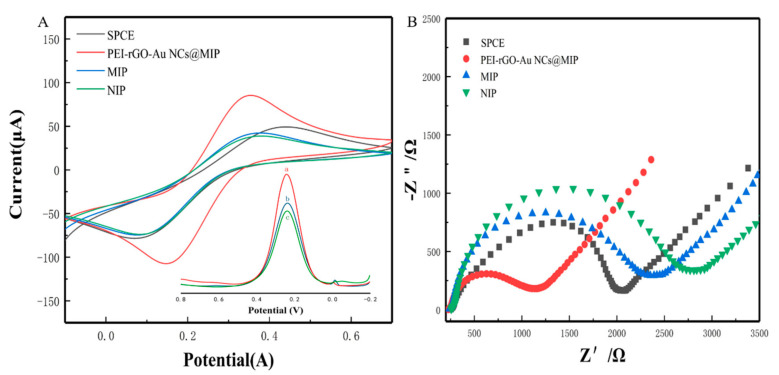
Characterization of the modified electrode. (**A**) CV curves (a: After washed; b: After incubation with β-Lg; c: Before washed); (**B**) EIS curves.

As shown in Figure 4A, a recoding of the cyclic voltammetry of [Fe(CN)_6_]^3−^ in a solution of 5.0 mmol/L K_3_[Fe(CN)_6_] containing 0.1 mol/L KCl. Different electrodes (bare SPCE, PEI-rGO-Au-NCs@MIP/SPCE, MIP/SPCE, NIP/SPCE) were compared by CV. As expected, the peak currents of MIP/SPCE and NIP/SPCE were lower than that of bare SPCE due to the poor conductivity of the MIP and NIP materials. The PEI-rGO-Au-NCs@MIP electrode exhibited the best electrochemical performance, which was attributed to the good electrical conductivity of PEI-rGO-Au-NCs. The DPV response curves before and after elution were shown in the illustration. From the DPV response curves, it can be seen that the material has better electrochemical performance after elution. 

Next, an electrochemical impedance spectroscope was used to evaluate the electron transfer behavior of the sensor. The surface resistance of the sensor was directly proportional to the diameter of the semicircle [12]. As shown in Figure 4B, modification with MIP or NIP increased the surface resistance compared to the bare electrode. The surface resistance of the electrode modified with PEI-rGO-Au-NCs@MIP was smaller than that of the bare electrode, because both the reduced GO and gold nanoclusters in PEI-rGO-Au-NCs@MIP can amplify the electric signal. After removing the template molecules, the cavities in PEI-rGO-Au-NCs@MIP emerged. Therefore, potassium ferricyanide reached the electrode surface through the cavities and the resistance value decreased.

### 3.4. Optimization Conditions for Electrochemical Analysis

**Figure 5:**
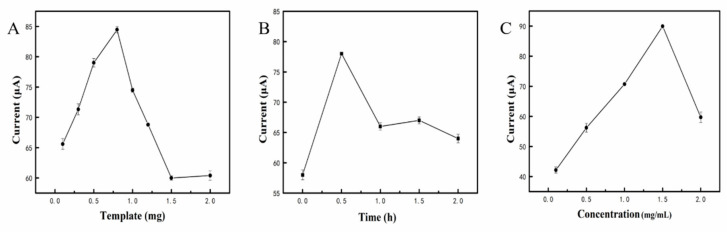
The influence of different factors on the performance of electrochemical sensor to detect β-Lg: (**A**): Template dosage; (**B**): Elution time; (**C**): Material concentration.

To obtain better electrochemical analysis results, for β-Lg, optimization experiments were carried out. In this study, the influence of the template dosage, elution time and material concentration were considered. Too little template will lead to a low current response, and too much template will make it difficult to form MIPs. As shown in Figure 5A, when the template dosage was 0.8 mg, there was a significant increase in current, so we used this dosage for further experiments. The influence of the elution time was also investigated, and the highest current response appeared after 30 min of elution (Figure 5B). As shown in Figure 5C, when the material concentration was increased to 1.5 mg/mL, the current response increased to 89 μA. After that, the current response showed a downward trend.

### 3.5. Electrochemical Behavior of the Modified Sensor

**Figure 6:**
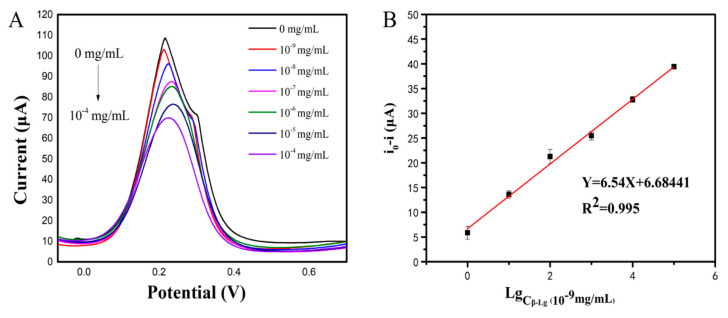
Analytical performance of the electrochemical sensor (**A**): DPV responses of the electrochemical sensor after incubation with different concentrations of β-Lg; (**B**): The calibration curve of the electrochemical sensor.

The analytical ability of the modified sensor was tested under the optimal conditions, with different concentrations of β-Lg. Figure 6A showed the differential pulse voltammetry (DPV) current response of the electrochemical sensor. The DPV response gradually decreased with the increase in β-Lg concentration. The reason may be that a large number of biomolecules affected the electron transfer efficiency. Figure 6B showed the calibration curve of the β-Lg concentration. It can be seen that there was a good linear relationship between the anode peak current value and β-Lg concentration. The linear regression equation is Y = 6.68441 + 6.54X, with a correlation coefficient (R^2^) of 0.995, and LOD of 10^−9^ mg/mL (S/N = 3). The detection range was 10^−9^ to 10^−4^ mg/mL. Obviously, the electrochemical sensor had a considerably lower detection limit and a wider linear range compared to the purchased ELISA kit (LOD = 0.02 mg/mL).

### 3.6. Reproducibility and Specificity of the Sensor

**Figure 7:**
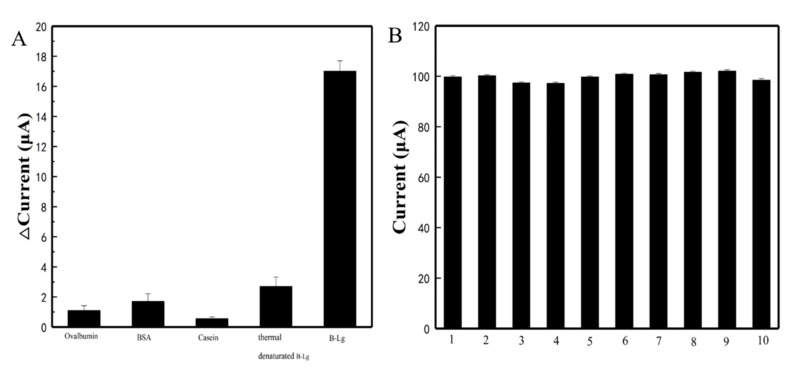
Evaluation of the performance of the electrochemical sensor (**A**): Specificity; (**B**): Reproducibility.

As possible interfering proteins commonly found in food, egg albumin, BSA, and casein were considered for the test. To study the specific recognition of β-Lg by PEI-rGO-Au-NCs@MIP/SPCE, egg albumin, BSA, casein and thermally denatured β-Lg were introduced to test the response currents. As shown in Figure 7, no significant change in current signal was observed after the addition of interfering substance at a concentration of 4 × 10^−^^8^ mg/mL, confirming the specificity of MIP on the target molecule structure. We attributed this specificity to the successful formation of imprinted cavities in the polymer matrix that were complementary in size, conformation, and shape to the target β-Lg molecule. These candidate interfering molecules were structurally different from β-Lg so that they cannot diffuse or bind strongly to the recognition sites and, thus, produce a very weak response. Moreover, reproducibility was an important characteristic of the feasibility of practical application of the modified electrode. We investigated the reproducibility of PEI-rGO-Au-NCs@MIP/SPCE by preparing the same PEI-rGO-Au-NCs@MIP/SPCE to detect β-Lg. The RSD of the response currents of ten different electrodes was 2.2%, revealing good reproducibility of PEI-rGO-Au-NCs@MIP/SPCE. These results indicated that the detection of β-Lg by PEI-rGO-Au-NCs@MIP/SPCE had good reproducibility and specificity.

### 3.7. Analysis of Real-World Samples

The proposed PEI-rGO-Au-NCs@MIP/SPCE sensor was applied to the determination of β-Lg in four milk samples (Jingdian, Telunsu, Yili, Mengniu) to demonstrate its practical application. Additionally, ELISA was used for comparison. As shown in Table 1, the measurement results of the electrochemical sensors were similar to those of ELISA, implying that the sensors can be conventionally used for the measurement of β-Lg. In addition, the detection limit of the ELISA was at least 20 μg/mL, while the detection limit of the electrochemical sensors was 10^−9^ mg/mL, which was much lower than that of the ELISA. These results prove that our proposed electrode detection method is sufficiently accurate for the detection of β-Lg in real milk samples.

### 3.8. Comparison with Other Electrochemical Detection Methods

Table 2 summarized the comparison of our electrochemical detection methods with other electrochemical methods in the detection of β-Lg. Compared with other electrochemical methods, the detection limit of our sensor was lower and the linear range was wider. Most importantly, the specificity of MIP reduced the interference of other substances in milk. Therefore, our method had unique advantages for practical applications.

## 4. Conclusions

In this study, a PEI-rGO-Au-NCs@MIP electrode was successfully fabricated and utilized in the electrochemical detection of β-Lg. Owing to the excellent conductivity of PEI-rGO-Au-NCs and the specific recognition ability of MIP, the PEI-rGO-Au-NCs@MIP biosensors exhibited high sensitivity and selectivity in the recognition of β-Lg. Furthermore, the obtained electrochemical sensor was successfully applied in the determination of β-Lg in real-world samples, and the results were similar to a commercial ELISA kit, which is considered as reliable analysis. The expensive β-Lg antibody from our previous research [27] was replaced with MIP, which significantly increased the selective surface area of PEI-rGO-Au-NCs@MIP and improved its sensitivity by providing a large number of imprinted sites, which resulted in a low detection limit (LOD = 10^−9^ mg/mL) and a wide linear range (10^−9^ to 10^−4^ mg/mL). In addition, MIP transformation by changing the template molecule may provide a new strategy for the development of electrochemical sensors, not only for β-Lg, but also for other substances.

## Figures and Tables

**Table 1:** Comparison of β-Lg detection in real samples using the electrochemical sensors and ELISA.

Sample	ELISA (*μ*g/mL)	sensor (*μ*g/mL)
Jindian	11.46 ± 0.17	11.74 ± 0.47
Telunsu	16.91 ± 0.47	17.00 ± 0.21
Yili	16.91 ± 0.42	17.01 ± 0.36
Mengniu	15.13 ± 0.24	14.94 ± 0.52

**Table 2:** Comparison with other detection methods.

Detection Methods	Sensor	Linearity Range (mg/mL)	LOD (mg/mL)	Reference
SPR		4.9 × 10^−4^–1	1.6 × 10^−4^	[36]
		10^−6^–5 × 10^−3^	3 × 10^−6^	[37]
ELISA		3.1 × 10^−5^–8 × 10^−3^	1.9 × 10^−6^	[38]
		4.8 × 10^−7^–6.3 × 10^−5^	4.9 × 10^−7^	[39]
Electrochemical methods	AuNPs@PLL/GSPEs	10^−7^–10^−3^	9 × 10^−8^	[40]
	PGE/ITO	0.53–11.2	0.27	[41]
	BiVO_4_/BiOBr@Ag_2_S/ITO	10^−8^–10^−4^	3.7 × 10^−9^	[42]
	PANI-PAA/GSPEs	10^−5^–10^−3^	5.3 × 10^−5^	[5]
	PEI-rGO-Au-NCs/SPEs	10^−8^–10^−4^	10^−8^	[27]
	PEI-rGO-Au-NCs@MIP/SPCE	10^−9^–10^−4^	10^−9^	This work

## Data Availability

The data presented in this study are available in this article.

## Abbreviations

SPR	Surface plasmon resonance
ELISA	Enzyme-linked immunosorbent assay
PLL	Poly-L-Lysine
GSPEs	Graphite Screen-Printed Electrodes
PGE	Pencil Graphite Electrode
ITO	Indium Tin Oxide
PANI/PAA	Poly(aniline-co-anthranilic acid)
SPEs	Screen-Printed Electrodes
SPCE	Screen-printed carbon electrode
